# Use of autologous tooth-derived material as a graft in the post-extraction socket. Split-mouth study with radiological and histological analysis

**DOI:** 10.1186/s12903-024-04576-0

**Published:** 2024-07-23

**Authors:** H. López Sacristán, M. del Canto Pingarrón, M.A. Alobera Gracia, J. de Elío Oliveros, R. Díaz Pedrero, J. Seco-Calvo

**Affiliations:** 1 Master’s in Oral Surgery, Implant Dentistry, and Periodontics, University of León, Avenida de Valladolid 2, Primera Planta Oficina 3., Aldeamayor de San Martin, Valladolid 47162 Spain; 2 Head of the Master’s programme, Master’s in Oral Surgery, Implant Dentistry, and Periodontics, University of León, León, Spain; 3 Department of Morphological Sciences and Surgery, School of Medicine, University of Alcalá, Madrid, Spain; 4 Institute of Biomedicine (IBIOMED), University of León, León, Spain

**Keywords:** Alveolar preservation, Autologous dentin graft, Post-extraction socket, Dimensional changes, Dentin, Histology

## Abstract

**Background:**

The healing process after tooth removal involves bone remodelling which implies some loss of alveolar bone volume. Among materials proposed for minimising this remodelling and preserving the bone, autologous dental tissue is a promising option, but more data are needed. In this context, we evaluated size and density changes using cone beam computed tomography in autologous dental material (ADM)-preserved sockets compared to controls, and assessed biological responses by histological analysis.

**Methods:**

A split-mouth study was conducted including 22 patients, who underwent removal of ≥ 2 single-rooted teeth with intact sockets, assigning one socket to the experimental group which received ADM for alveolar preservation and another to the control group, which only underwent blood clot stabilisation. Cone beam computed tomography was performed postoperatively (week 0) and at weeks 8 and 16 to assess socket size and bone density. Histological analysis was carried out on trephine biopsies taken (Ø4 × 4.5 mm) from the experimental group.

**Results:**

Less horizontal shrinkage was observed in the ADM group, especially at week 16 considering the group-by-time interaction for the following variables: difference in height between the lingual and buccal alveolar crests (-1.00; *p* < .01; 95% CI: -0.28 – -1.73), and half-widths, measured as the distance from the long axis of the missing tooth to the buccal alveolar crest at 1 mm (-0.61; *p* < .01; 95% CI: -0.18 – -1.04) and at 3 mm (-0.56; *p* < .01; 95% CI: -0.15 – -0.97) below the crest, with mean decreases of 1.07 and 2.14 mm in height difference, 0.66 and 1.32 mm in half-width at 1 mm and 0.43 and 1.02 mm in half-width at 3 mm in ADM and control groups respectively. Densitometry analysis showed higher bone densities in Hounsfield units in the ADM group considering all factors analysed regardless of time point and socket third (coronal, middle, or apical). Histologically, there were no signs of inflammation or foreign body reaction, and dentin particles were surrounded by and in close contact with bone tissue.

**Conclusion:**

These results add to the evidence that dentin can be used successfully as a material for alveolar socket preservation, given its desirable mechanical and biological properties, and warrant larger studies.

## Background

Bone resorption is a phenomenon that occurs naturally as part of physiological remodelling. Cells called osteoclasts break down the bone matrix, while osteoblasts work to form new bone. To maintain the structural integrity of bone, there must be a balance between bone formation and resorption. In the jaw, this balance can be disrupted after a tooth extraction, potentially leading to greater resorption than formation, especially in the walls of the tooth socket. Substantial resorption of the alveolar crest after tooth extraction has been documented in both experimental animal models and humans (e.g., 1–3). This can have significant implications for the planning of dental prostheses or subsequent dental implant placement [4, 5].

The healing process that begins following tooth extraction is complex, involving several physiological changes in the alveolar bone tissue. Immediately after extraction, a blood clot forms in the socket, this being essential for protecting the underlying bone and serving as a matrix for the invasion of reparative cells. During the first few weeks, the clot is gradually replaced by granulation tissue and then by newly formed bone tissue. The literature suggests that numerous factors can influence this process, including patient age, whether there is pre-existing periodontal disease, and the surgical technique used for extraction [1–3]. Therefore, there is a need to carefully assess the extraction site and employ techniques that promote bone preservation [6]. In modern dentistry, alveolar ridge preservation is considered essential, particularly following tooth extraction, to maintain the integrity and aesthetics of the dental alveoli [7]. The systematic review conducted by Avila Ortiz in 2018 [3] underscores the importance of alveolar ridge preservation techniques and their significant impact on the long-term outcomes of dental implants. Assessing the efficacy of different preservation methods, these authors suggest that the choice of method should be based on a careful evaluation of the individual case, considering factors such as the anatomy of the extraction site as well as patient expectations.

Another factor to be considered is the activity of myofibroblasts, given the current evidence that they are partly responsible for the extensive bone remodelling observed following an extraction [8]. Myofibroblasts are specialized cells that play a crucial role in tissue repair, in particular, in wound contraction and closure. Nonetheless, their persistent activation can lead to pathological fibrosis, resulting in excessive scarring and organ dysfunction. Given this, inhibition of myofibroblast activity represents a potential therapeutic approach to prevent or reduce fibrotic diseases and promote wound healing, though research findings have yet to be translated to clinical practice [9, 10].

Further, to minimize the impact of bone remodelling, the literature suggests various options which include reducing trauma and infection risk associated with extraction procedures [11, 12], using customised healing abutments [13], and facilitating primary wound closure and healing using grafts, membranes and fillers such as collagen, plasma derivatives and/or bone substitutes [14–18]. To maintain the socket integrity as far as possible, various tooth substitutes may be used to provide appropriate mechanical support. The ideal characteristics of these substitutes have been well described: biocompatibility, osteoconduction, and biodegradability, as well as absorbability allowing replacement by the patient´s own bone [19–21], though the concept of replacement has been questioned, it having been shown that slow resorption or even permanence of filler particles may be beneficial for the long-term maintenance of the tooth socket [22, 23].

Autologous dental tissue is considered an interesting material for stimulating bone regeneration. Dentin represents 85% of the structure of the tooth, is an easily accessible resource, and offers a higher mineral content than any other bone-derived material [24]. It is comparable to autologous bone tissue in at least two characteristics, namely, osteocompatibility, and osteoconduction, and hence could provide a matrix for bone neoformation [25–27]. Further, recent studies have suggested that dentin has proteins that are common to bone and tooth root cementum. These may favour bone formation and calcification as their composition is similar to that of bone tissue, both being derived from neural crest cells and composed of the same type of collagen (type I) [26]. In particular, dentin contains bone morphogenetic proteins (BMPs), which induce bone formation, and non-collagenous proteins such as osteocalcin, osteonectin, and phosphoprotein, which are involved in bone calcification. Similar to bone, between 70 and 75% of dentin is inorganic, while organic matter accounts for approximately 20%, and at least 90% of this organic matter is collagen type I [26]. There is already some evidence that dentin could play a useful role in early bone repair and provide a suitable surface for osteoblast adhesion and proliferation (e.g., [27]).

Murata´s research team, having reported the first case of autologous dentin grafting in 2003 (Murata, M. Autogenous demineralized dentin matrix for maxillary sinus augmentation in human. The first clinical report. 81st International Association for Dental Research,2003, Goteborg, Sweden, 2003, June), presented dentin as a new biomaterial and a BMP-rich matrix (BMP-2) for bone regeneration in humans in 2011 [28]. They noted that early studies in rabbits had found that bone formation was induced within 4 weeks after grafting using completely demineralised dentin matrix, and within 8–12 weeks using non-demineralised dentin [29]. After dentin demineralisation, some types of BMPs, in particular, BMP-2, -4, and − 7, are still bioactive and these bind in collagen-rich matrices, as in bone. On the other hand, the delay in the case of using non-demineralised material may be due to an inhibition of BMP release by apatite crystals. Despite this potentially slower bone formation associated with the use of less processed dentin-based material, freshly ground dentin can be considered a good candidate as a biomaterial for use in the preservation and regeneration of bone tissue in clinical practice, given its biological characteristics and availability (e.g., [30]) and there is some evidence of its efficacy in alveolar ridge preservation in humans (e.g., [31, 32]).

Given all this, the objective of our research was to conduct a split-mouth study to compare the use of fresh ground dentin (ADM) as a filler with that of blood clot stabilisation alone in terms of alveolar ridge preservation. Our hypothesis was that the use of ADM for socket preservation would decrease bone remodelling after tooth extraction.

## Methods

**Fig. 1:**
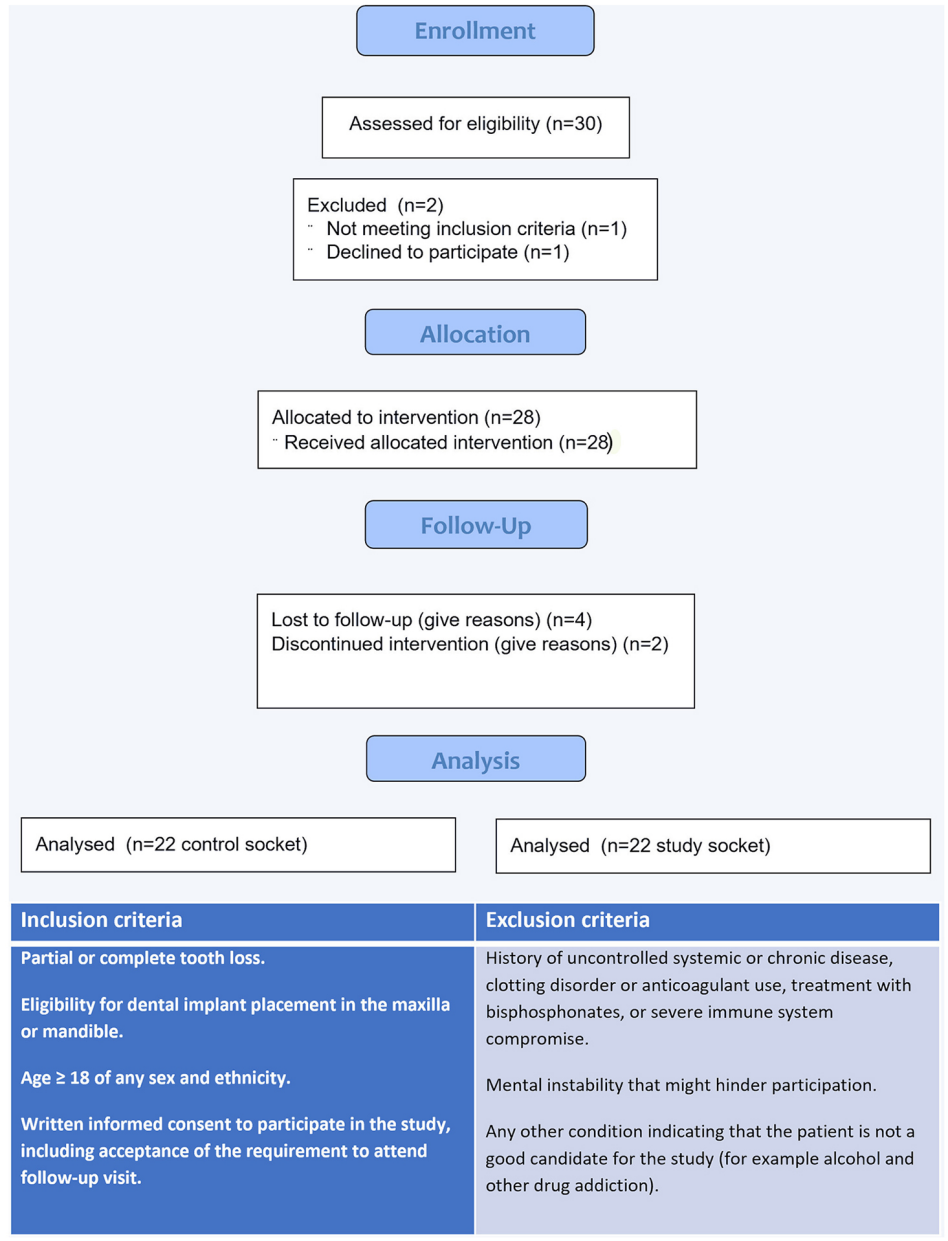
Flow of patients through the study including selection criteria

This clinical study was conducted in the facilities of the Master’s in Oral Surgery, Implant Dentistry, and Periodontics at the University of León, building on a pilot study on the use of ADM for alveolar grafting after tooth extraction conducted by our research group [33]. The selection criteria and flow of patients through the study are presented in Fig. 1.

Before enrolment, patients were provided with information about the study and alternative treatments, given an opportunity to ask questions concerning the study, and then asked to sign an informed consent form declaring that they were aware of the scope of the research (including surgical interventions and associated risks). This study was approved by the Ethics Committee of the University of León (Ref. no. ETICA-ULE-034-2018) and has been conducted in accordance with its strict criteria and the principles of the Declaration of Helsinki.

### Study design

Once patients had been included, they underwent the removal of soft tissue (syndesmotomy), and any teeth that could not be saved or that were deemed likely to have an insignificant or counterproductive role in aesthetic and functional rehabilitation outcomes. Further, teeth were removed from sites deemed suitable for implants. For this study, we selected two non-adjacent sockets of single-rooted teeth, if possible, at contralateral sites, and if not, at sites in the corresponding maxillary or mandibular half with similar structural conditions, and in all cases, with no damage to the bony walls. One socket was assigned to the study group, and this received alveolar ridge preservation with autologous dental material (ADM). The ADM was covered with a collagen membrane (Lyoplant, B. Braun, Hessen, Germany) secured with monofilament 5 − 0 sutures (Anclalon Nylon Azul DRT12, Ancladén, Barcelona, Spain) in line with recent recommendations [7]. The material used was obtained from the patient’s own extracted teeth, which were crushed, converted into particles, and disinfected, following the instructions for the Kometabio^®^ Smart Dentin Grinder processing system. The other socket was assigned to the control group, and underwent just blood clot stabilisation, with the same type of collagen membrane secured with 5 − 0 monofilament sutures.

For both groups, cone beam computed tomography (CBCT) of the sockets was performed immediately after surgery (W0), at 8 weeks (W8), and finally, at 16 weeks (W16). At this last time point, if considered necessary for implant planning, samples were collected with a trephine drill (4- and 4.5-mm internal and external diameter, respectively).

### Radiological assessment

The CBCT scans were taken in the facilities of the Master’s in Oral Surgery, Implant Dentistry, and Periodontics at the University of León (CS 9300 system, Carestream Health, Rochester, NY). The radiation dose was adjusted for body weight (591, 685, and 856 mGy/cm^2^ for body weights < 60, between 60 and 90, and > 90 kg, respectively). The computed tomography analysis was performed using BTI Scan 3 software (BTI Biotechnology Institute, Vitoria, Araba, Spain).

**Fig. 2:**
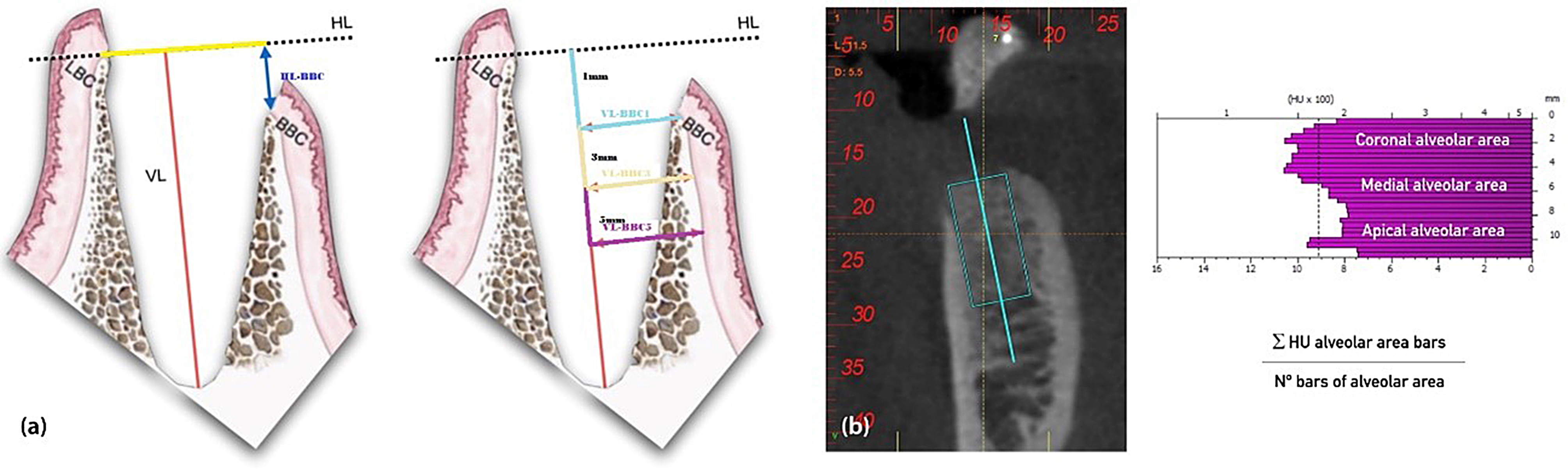
Schematic diagram of dimensional measurements taken. (**a**) and calculation of bone density in Hounsfield units (**b**) Legend: HL: horizontal line (yellow); VL: vertical line (red); BBC, BBC1, BBC3 and BBC5: buccal cortical bone and at 1, 3 and 5 mm from the crest respectively; W0, W8 and W16: baseline and weeks 8 and 16 respectively Adapted from Canto-Díaz, A., De Elio-Oliveros, J., Del Canto-Díaz, M., Alobera-Gracia, M.A., Del Canto-Pingarrón, M., Martínez-González, J.M., 2019. Use of autologous tooth-derived graft material in the post-extraction dental socket. Pilot study. Med. Oral Patol. Oral Cir. Bucal 24 [1], e53–e60. 10.4317/medoral.22536

Images were analysed using the method designed for the pilot study [33] and in line with methods used by other authors for similar analysis [34] (Fig. 2).

#### Dimensional analysis of the tooth socket

We measured the following dimensional parameters in CBCT images at all time points (W0, W8, and W16), as illustrated in Fig. 2 (a):

##### Vertical measurements

socket height, measured vertically along the long axis of the missing tooth from the fundus of the socket to the lingual alveolar crest (VL, red vertical line); and difference in height between the lingual and buccal alveolar crests, measured as the minimum distance from a line drawn horizontally at the lingual crest to a perpendicular line from the outer part of the vestibular cortex (HL-BBC, dark blue line).

##### Horizontal measurements

socket width, measured as the distance along the aforementioned horizontal line from the lingual alveolar crest to the aforementioned perpendicular line from the buccal crest (HL, yellow line); and half-widths measured from the VL to the BBC at 1 mm (pale blue line), 3 mm (beige line), and 5 mm (purple line) below the crest (VL-BBC1, VL-BBC3, and VL-BBC5, respectively).

#### Densitometry analysis of the tooth socket

Bone density of the regenerated bone in the post-extraction socket was analysed by measuring the mean density in Hounsfield units (HUs) as in other recent studies assessing implant site bone quality [35, 36]. We measured the density in the coronal, medial, and apical thirds of the socket immediately after the surgery and at 8 and 16 weeks using the equation described in the pilot study. In brief, the total number of bars shown in Fig. 2 was divided by three, to obtain the number of bars in each third of the socket (denominator), and the sum of the HUs across all the bars in each third was used as the numerator.

### Histological assessment

Samples collected were fixed with 10% formaldehyde solution and buffered to a pH of 7 to avoid decalcification, and subsequently sent to the Microscopic Anatomy Laboratory (Human Anatomy Unit, Department of Surgery, Medical and Social Sciences, University of Alcalá, Madrid, Spain), for further processing according to the following protocol: (1) Replacement of formaldehyde by polymethyl methacrylate using increasing concentrations, and subsequently, of polymethyl methacrylate by liquid methacrylate, which solidifies by photopolymerisation, becoming sufficiently hard to allow histological sections to be taken; (2) Taking of sections without decalcifying using the Exakt system, obtaining four or five sections transverse to the longitudinal axis of the sample, yielding a larger number of sections than would be possible following the longitudinal axis; (3) Staining of sections with toluidine blue; (4) Histological analysis, including type of bone tissue and the presence of dentin in each section; and (5) Evaluation of the presence of an inflammatory or foreign body reaction.

### Sample size

Following the approach adopted by authors of similar research [37], a post hoc sample size calculation was performed with the G*Power tool (version 3.1.9.7) (https://www.psychologie.hhu.de/arbeitsgruppen/allgemeine-psychologie-und-arbeitspsychologie/gpower). With at least 22 cases in the experimental group (which received ADM) and 22 cases in the control group (which only underwent blood clot stabilisation), it was estimated that the study would have a power of 0.82 to detect between-group differences with an effect size d of 0.8 and an alpha error of 0.05. We considered this sufficient for the purposes of this study, and to allow for losses, initially recruited 30 patients.

### Statistical analysis

Statistical analysis was performed using IBM-SPSS Statistics, version 25. Qualitative variables were described with frequency tables and percentages. Quantitative data were explored to assess whether they were normally distributed using: (a) normal Q-Q plots, (b) measures of skewness and kurtosis, and (c) the Shapiro-Wilk goodness of fit tests (for sample sizes, *N* < 50) to assess normality, where only a large deviation (*p* < .1) would make us consider that the variable is not normally distributed. Quantitative variables were described using the usual measures of (a) central tendency: mean and median; and (b) variability: observed range, standard deviation, and interquartile range.

Student’s t-tests were employed for comparing the means of independent samples for normally distributed variables.

Two-factor analysis of variance for related samples was used for comparing the means of measurements from different time points when data were normally distributed. Effect sizes were calculated, to express the magnitude of the differences between samples, and expressed as R^2^ (from 0 to 1). When means were compared, R^2^ was calculated using Cohen’s d. For the inferential analysis, *p* < .05 was considered significant and *p* < .01 highly significant, while *p* < .10 was considered to indicate a tendency to significance or close to significance (< 10%).

## Results

During the study, six patients withdrew or were excluded from the programme due to failing to attend scheduled appointments, and two were excluded after being diagnosed with systemic conditions not compatible with this research. Finally, a total of 22 patients were included.

### Radiological results

We collected data on a total of 44 sockets, 22 in the half mouth used for the study group (ADM group) and 22 in that used for the control group. Measurements of the variables of interest were taken at three time points (W0, W8, and W16), allowing repeated measures analysis.

The data were analysed for each half-mouth separately (Tables 1 and 2). In general, statistical analysis, including Q-Q plots, indicated that the data were normally distributed. In cases of deviation, although significant (*p* < .05), the extent of misfit can be considered slight, with a tendency to normality. Therefore, we opted to apply parametric statistical tests in the following between-group and between-time point comparisons (Tables 1 and 2). Specifically, we compared the mean values of the nine parameters of interest as a function of the two study factors: time point (repeated measures at three time points), and related sample/group (ADM and control groups).

**Table 1:** Descriptive statistics for the control group (*N* = 22)

Variables		Test: distribution			Central tendency		Range	Dispersion	
		Skewness	Kurtosis	Shapiro-Wilk *p*-value	Mean	Median		Standard deviation	Interquartile range
*Socket size*	*HL – W0*	.61	.40	.329 ^NS^	8.58	8.25	5.7–11.9	1.50	1.88
	*VL – W0*	.58	1.25	.485 ^NS^	11.90	12.15	9.3–15.8	1.45	1.70
	*HL-BBC – W0*	.55	.04	.531 ^NS^	1.57	1.40	.0–4.1	1.04	1.48
	*VL-BBC1 – W0*	.36	.74	.537 ^NS^	4.22	4.30	2.8–6.0	.74	.75
	*VL-BBC3 – W0*	.93	1.60	.212 ^NS^	4.00	3.95	2.7–6.1	.77	.83
	*VL-BBC5 – W0*	.95	1.04	.093 ^NS^	3.90	3.90	2.7–6.4	.92	1.20
	*HL – W8*	1.10	.79	.024*	7.02	6.70	5.1–11.4	1.72	2.55
	*VL – W8*	.44	.03	.415 ^NS^	10.78	10.50	8.7–14.1	1.41	1.95
	*HL-BBC – W8*	2.91	1.52	.000*	2.39	1.90	.7–10.5	2.11	1.93
	*VL-BBC1 – W8*	1.41	3.80	.021*	3.30	3.25	2.0–6.0	.85	.90
	*VL-BBC3 – W8*	1.98	5.04	.001*	3.25	3.10	2.3–6.2	.89	.88
	*VL-BBC5 – W8*	1.47	2.86	.011*	3.40	3.05	2.2–6.6	1.05	1.20
	*HL – W16*	1.40	2.27	.017*	6.44	6.00	4.1–11.4	1.74	1.93
	*VL – W16*	.53	.68	.449 ^NS^	10.16	10.10	7.6–14.1	1.61	2.13
	*HL-BBC – W16*	.46	− .43	.622 ^NS^	1.84	1.65	.1–4.1	1.02	1.53
	*VL-BBC1 – W16*	1.93	5.93	.002*	2.90	2.80	1.9–5.7	.81	1.03
	*VL-BBC3 – W16*	1.71	3.96	.004*	2.98	2.85	1.9–5.7	.85	1.03
	*VL-BBC5 – W16*	1.32	2.66	.032*	3.22	2.90	1.8–6.4	1.06	1.10
*Bone density in Hounsfield units*	*CORONAL – W0*	.40	.13	.538 ^NS^	253.47	257.70	.0–652.7	169.14	230.53
	*MEDIAL – W0*	− .16	− .49	.643 ^NS^	316.23	355.50	.0–693	185.35	284.00
	*APICAL – W0*	− .84	− .41	.012*	385.85	437.75	.0–598.8	186.65	337.63
	*CORONAL – W8*	.95	.30	.071 ^NS^	479.52	421.05	173.3–1050.5	241.99	299.98
	*MEDIAL – W8*	.97	1.50	.049*	520.27	529.50	224.0–978	191.76	211.50
	*APICAL – W8*	.44	.44	.857 ^NS^	539.43	508.55	169.4–960.5	187.55	219.60
	*CORONAL – W16*	.96	1.00	.082 ^NS^	645.41	545.25	204.4–1376.1	297.38	373.83
	*MEDIAL – W16*	.50	1.02	.870 ^NS^	650.65	619.75	171.5–1256.0	236.42	298.88
	*APICAL – W16*	.05	− .21	.986 ^NS^	690.05	692.70	262.2–1134.4	222.09	327.03

NS = non-significant deviation (*p* > .05), the data are normally distributed. * = significant but slight deviation (*p* < .05); the data tend to a normal distribution

HL: horizontal line; VL: vertical line; BBC, BBC1, BBC3 and BBC5: buccal cortical bone crest and at 1, 3 and 5 mm from the crest respectively; W0, W8 and W16: baseline and weeks 8 and 16 respectively. See also Fig. 2

**Table 2:** Descriptive statistics for autologous dental material group (*N* = 22)

Variables		Test: distribution			Central tendency		Range skewness	Dispersion	
		Skewness	Kurtosis	Shapiro-wilk *p*-value	Mean	Median		Kurtosis	Shapiro-wilk *p*-value
	*HL – W0*	1.37	3.24	.036*	8.51	8.25	6.6–12.8	1.38	1.73
*Socket size*	*VL – W0*	.66	.17	.491 ^NS^	11.96	11.55	9.1–16.8	1.97	2.93
	*HL-BBC – W0*	1.75	5.54	.005*	1.51	1.40	.0–5.3	1.12	1.10
	*VL-BBC1 – W0*	.06	-1.13	.184 ^NS^	4.17	4.10	3.2–5.4	.65	1.08
	*VL-BBC3 – W0*	− .77	1.61	.323 ^NS^	3.97	4.00	2.0–5.2	.71	1.05
	*VL-BBC5 – W0*	.00	-1.03	.276 ^NS^	3.72	3.80	2.8–4.7	.58	.92
	*HL – W8*	2.14	6.83	.001*	7.80	7.80	6.2–12.6	1.36	1.58
	*VL – W8*	1.32	3.35	.049*	11.06	10.90	8.8–15.8	1.53	1.60
	*HL-BBC – W8*	1.31	4.47	.015*	1.38	1.45	.0–3.9	.78	.80
	*VL-BBC1 – W8*	− .60	.51	.271 ^NS^	3.66	3.75	2.2–4.7	.62	.83
	*VL-BBC3 – W8*	− .87	.85	.178 ^NS^	3.69	3.80	2.1–4.6	.60	.80
	*VL-BBC5 – W8*	− .58	.14	.395 ^NS^	3.54	3.70	1.8–4.7	.72	.95
	*HL – W16*	2.07	7.00	.001*	7.44	7.05	5.3–12.6	1.46	1.68
	*VL – W16*	1.54	3.62	.015*	10.75	10.40	8.7–15.7	1.56	1.75
	*HL-BBC – W16*	1.41	4.51	.020*	1.29	1.20	.0–3.7	.74	.85
	*VL-BBC1 – W16*	− .43	− .11	.286 ^NS^	3.51	3.60	2.2–4.5	.63	.90
	*VL-BBC3 – W16*	− .54	.69	.424 ^NS^	3.54	3.60	2.0–4.5	.60	.75
	*VL-BBC5 – W16*	− .21	− .35	.863 ^NS^	3.44	3.60	2.0–4.6	.67	.93
*Bone density in Hounsfield units*	*CORONAL – W0*	.40	-1.22	.047*	1282.14	1242.50	1017.2–1567.7	178.70	304.73
	*MEDIAL – W0*	− .05	-1.25	.170 ^NS^	1305.39	1351.25	1058.0–1589.0	160.08	303.25
	*APICAL – W0*	-1.06	2.69	.067 ^NS^	1210.75	1214.15	547.2–1571.1	220.13	334.93
	*CORONAL – W8*	-1.39	2.57	.024*	1152.74	1172.95	512.7–1456.1	215.90	227.18
	*MEDIAL – W8*	− .13	.58	.612 ^NS^	1268.05	1239.75	911.5–1551	148.60	145.50
	*APICAL – W8*	.52	.16	.581 ^NS^	1158.85	1146.90	925.0–1432.2	127.25	147.75
	*CORONAL – W16*	− .52	.17	.464 ^NS^	1270.23	1295.25	779.4–1597.7	210.11	231.53
	*MEDIAL – W16*	− .26	− .64	.369 ^NS^	1326.86	1305.25	893.5–1600.0	197.40	331.88
	*APICAL – W16*	-1.23	3.17	.075^NS^	1198.70	1204.95	478.3–1600.0	234.80	281.25

NS = non-significant deviation (*p*>.05), the data are normally distributed

* = significant but slight deviation (*p*<.05); the data tend to a normal distribution

HL: horizontal line; VL: vertical line; BBC, BBC1, BBC3 and BBC5: buccal cortical bone crest and at 1, 3 and 5 mm from the crest respectively; W0, W8 and W16: baseline and weeks 8 and 16 respectively. See also Figure 2

#### HL

**Fig. 3:**
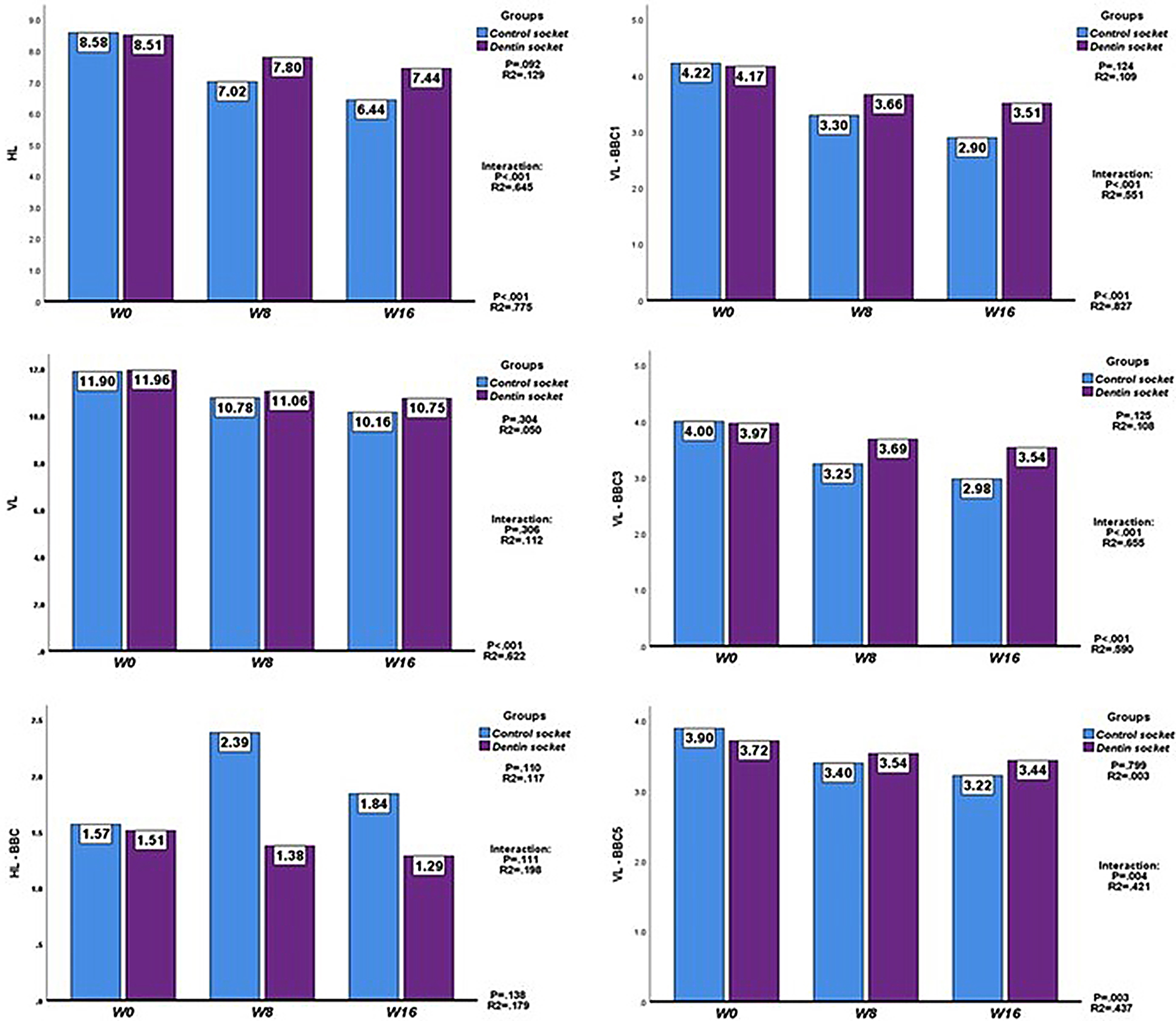
Plots comparing horizontal and vertical socket dimensions at each time point by split-mouth group. Legend: Results expressed as mean values. Statistics on the graph indicate the significance of between-group differences, group-by-time interactions, and between-time point differences, together with effect sizes HL: horizontal line; VL: vertical line; BBC, BBC1, BBC3 and BBC5: buccal cortical bone and at 1, 3 and 5 mm from the crest respectively; W0, W8 and W16: baseline and weeks 8 and 16 respectively. See also Fig. 2

We found highly significant differences (*p* < .01) and a very large size effect (R^2^ = 0.775) as a function of measurement time, this being robust statistical evidence to assert that the mean distance decreased over time (Fig. 3: later times associated with shorter distances). Comparing the two groups, the mean distance was almost the same at W0, while at W8 and W16, it had reduced less in the ADM group. Overall, we did not find a significant between-group difference, although it was close to significance (*p* < .100) with a moderate effect size (R^2^ = 0.129). Regarding the group-by-time interaction, we again found a highly significant difference (*p* < .001) with a very large effect size (R^2^ = 0.645). Hence, we can conclude that the reduction observed in time did differ between the groups, the difference being larger at W16 (-1.00; *p* < .01; 95% CI: -0.28 – -1.73) than at W8 (-0.78; *p* > .05; 95% CI: -0.03 – -1.52) (Table 3).

**Table 3:** Socket size and bone density at each time point by split-mouth group

Variable		**__________Week 0___________**		__________Week 8__________		_________Week 16_________	
		Controls	ADM group	Controls	ADM group	Controls	ADM group
	HL	8.58 (±1.50)	8.51 (±1.38)	7.02 (±1.72)	7.80 (±1.36)	6.44 (±1.74)	7.44 (±1.46)
	VL	11.90 (±1.45)	11.96 (±1.97)	10.78 (±1.41)	11.06 (±1.53)	10.16 (±1.61)	10.75 (±1.56)
Horizontal and vertical socket dimensions	HL-BBC	1.57 (±1.04)	1.51 (±1.12)	2.39 (±2.11)	1.38 (±.78)	1.84 (±1.02)	1.29 (±.74)
	VL-BBC1	4.22 (±.74)	4.17 (±.65)	3.30 (±.85)	3.66 (±.62)	2.90 (±.81)	3.51 (±0.63)
	VL-BBC3	4.00 (±.77)	3.97 (±.71)	3.25 (±.89)	3.69 (±.60)	2.98 (±.85)	3.54 (±0.60)
	VL-BBC5	3.90 (±.92)	3.72 (±058)	3.40 (±1.05)	3.54 (±.72)	3.22 (±1.06)	3.44 (±0.67)
	CORONAL	253.47 (±169.14)	1282.14 (±178.7)	479.52 (±241.99)	1152.74 (±215.9)	645.41 (±297.38)	1270.23 (±210.11)
Bone density in Hounsfield units	MEDIAL	316.23 (±185.35)	1305.39 (±160.08)	520.27 (±191.76)	1268.05 (±148.6)	650.65 (±236.42)	1326.86 (±197.4)
	APICAL	385.85 (±186.65)	1210.75 (±220.13)	539.43 (±187.55)	1158.85 (±127.25)	690.05 (±222.09)	1198.70 (±234.8)

Results expressed as means (with standard deviations) of each of the variables studied. N=22 per group

HL: horizontal line; VL: vertical line; BBC, BBC1, BBC3 and BBC5: buccal cortical bone and at 1, 3 and 5 mm from the crest respectively; W0, W8 and W16: baseline and weeks 8 and 16 respectively. See also Figure 2

#### VL

Similarly, we observed high significant differences with *p* < .001 and a very large effect size (R^2^ = 0.622) as a function of time, robust evidence to assert that the distance decreased with time (Fig. 3: later times, shorter distances). Further, the distance was almost identical in the two groups at W0, while at W8 and W16, it had decreased less in the ADM group. On the one hand, we did not find significant differences overall between groups (*p* > .05); and on the other, there was not a significant interaction (*p* > .05). Hence, it can be stated that the difference between the groups was similar at W8 (-0.28; *p* > .05) and W16 (-0.59; *p* > .05); although in this case, there was a moderate-to-large effect size (R^2^ = 0.112) which could be considered suggestive of a greater between-group difference in distance at W16 (Table 3).

#### HL-BBC

For this parameter, we did not find significant differences between time points (*p* > .05) or groups overall (*p* > .05), or a significant interaction (*p* > .05), indicating that the variations over time were similar in the two groups. Nonetheless, the effect sizes were moderate to high (R^2^ = 0.117 between groups) and even high between time points (R^2^ = 0.138) and for the interaction (R^2^ = 0.198), which could be considered suggestive of a relationship that might have reached statistical significance with a larger sample. Specifically, we note that while the mean distances are almost identical in the two groups at W0, by W8, there has been a marked increase in the control group and a slight decrease in the ADM group, the difference between groups reaching close to significance (1.01; *p* = .069; 95% CI: -0.09–2.10), and by W16, the mean distance in the control group had decreased to lower than at W8 and that in the ADM group had decreased further, the difference between groups again being close to significance (0.55; *p* = .053); 95% CI: -0.01–1.12). (Table 3)

#### VL-BBC1

We observed highly significant differences (*p* < .001) and a very large effect size (R^2^ = 0.827) between time points, allowing us to conclude that the mean distance decreases over time (Fig. 3; later times, shorter distances). In contrast, the overall between-group difference was not significant (*p* > .05) although the effect size was moderate (R^2^ = 0.109), suggestive of possible differences between groups. Considering the group-by-time interaction, we again found high statistical significance (*p* < .001) and a very large size effect (R^2^ = 0.551), enabling us to conclude that the reduction observed over time differed between the groups; and the difference was greater at W16 (-0.61; *p* < .01; 95% CI: -0.18 – -1.04) than at W8 when the difference was only close to significance (-0.37; *p* = .090; 95% CI: 0.06 – -0.80) (Table 3).

#### VL-BBC3

The pattern was the same as that seen for VL-BBC1. There were highly significant differences (*p* < .001) with a very large size effect (R^2^ = 0.590) between time points, robust statistical evidence for a decrease in this distance over time (Fig. 3; later times, shorter distances), more markedly in the controls than in the ADM group. In the overall comparison, the between-group difference was not significant (*p* > .05), but the effect size was moderate (R^2^ = 0.108), suggestive of potential differences between groups. Regarding the group-by-time interaction, we again observed high statistical significance (*p* < .001) and a very large effect size (R^2^ = 0.655), and hence, can conclude that the reduction over time differed between groups, and the difference was greater at W16 (-0.56; *p* < .01; 95% CI: -0.15 – -0.97) than at W8, at which point, the difference was again only close to significance (-0.44; *p* = .060; 95% CI: 0.02 – -0.90) (Table 3).

#### VL-BBC5

For this last parameter, neither the between-time point or between-group differences nor the interaction reached statistical significance (*p* > .05 in all cases), indicating that the changes over time were similar in the two groups. While the effect size was negligible for the between-group difference, it was very large for the between-time point comparison and the interaction (0.437 and 0.421 respectively). In the figure, we can see that while the distance was similar in the two groups at W0, it had decreased by W8 and W16 in both groups, though with larger reductions in the controls. The between-group differences at these later time points were not significant and were similar in value: -0.14 (95% CI: -0.65–0.37) at W8 vs. -0.21 (95% CI: -0.69–0.26) at W16. (Table 3)

#### Bone density in hounsfield units

The results were very similar for the three parameters considered. In the controls, the mean density was seen to increase from W0 to W16, while in the ADM group, the density decreased somewhat from baseline to W8, and then increased by W16 to values similar to those at W0. All the factors analysed (between-group and between-time point differences and their interaction) were significant (*p* < .01) or highly significant (*p* < .001), with very large effect sizes in all cases.

Specifically, in the **coronal** third of the bone, there were marked changes in density over time (*p* < .01 and R^2^ = 0.453) and between-group differences (*p* < .001 and R2 = 0.924), while their interaction indicated that the density varied differently over time in the two groups (*p* < .001 and R2 = 0.594). Between-group differences were highly significant (*p* < .001) at all time points: 1028.67 (95% CI: 917.95–1139.40), 673.21 (95% CI: 571.03–775.40), and 624.82 (95% CI: 506.33–743.31) HUs at W0, W8 and W16 respectively. Similarly, in the **medial** third of the bone, there were highly significant density changes over time (*p* < .001 and R^2^ = 0.578) and large overall between-group differences (*p* < .001 and R2 = 0.955), and again their interaction Indicated different patterns over time (*p* < .001 and R2 = 0.535). Further, between-group differences were highly significant (*p* < .001) at all time points: 989.16 (95% CI: 883.09–1095.23), 747.77 (95% CI: 655.88–839.88), and 676.21 (95% CI: 568.39–784.02) HUs at W0, W8 and W16 respectively. Lastly, in the **apical** third of the bone, once again, there were highly significant changes over time (*p* < .001 and R^2^ = 0.453), large overall between-group differences (*p* < .001 and R2 = 0.924), and an interaction indicating different density patterns over time (*p* < .001 and R2 = 0.594). Between-group differences were significant (*p* < .001) at all time points: 824.90 (95% CI: 709.57–940.22), 619.43 (95% CI: 525.66–713.19), and 508.65 (95% CI: 395.13–622.17) HUs at W0, W8, and W16 respectively. (Table 3)

### Histological findings

In the histological analysis, dentin particles were observed in all the samples taken from the ADM group at 16 weeks after implantation. On the other hand, we detected no signs of inflammation or foreign body reaction in any of the samples, indicating good biocompatibility of the dentin-based material used for alveolar ridge preservation.

**Fig. 4:**
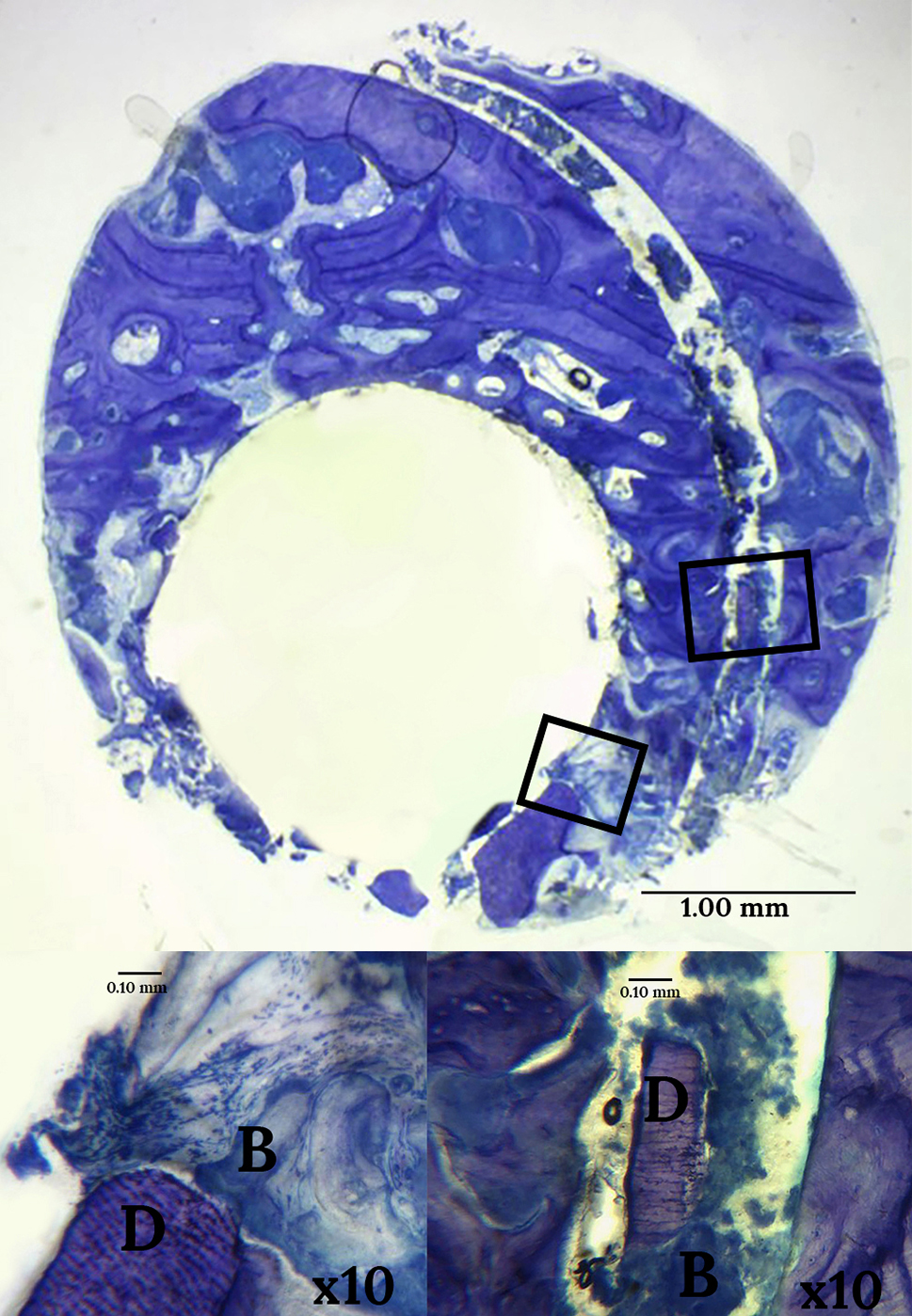
Histological images showing dentin particles in contact with bone. Legend: Cross-section. ADM side. Toluidine blue staining. 3x magnification. The bone is observed in blue In the magnified images (10x), we can observe dentin particles (**D**) in close contact with a fragment of newly formed bone (**B**) which contains lacunae of osteocytes

**Fig. 5:**
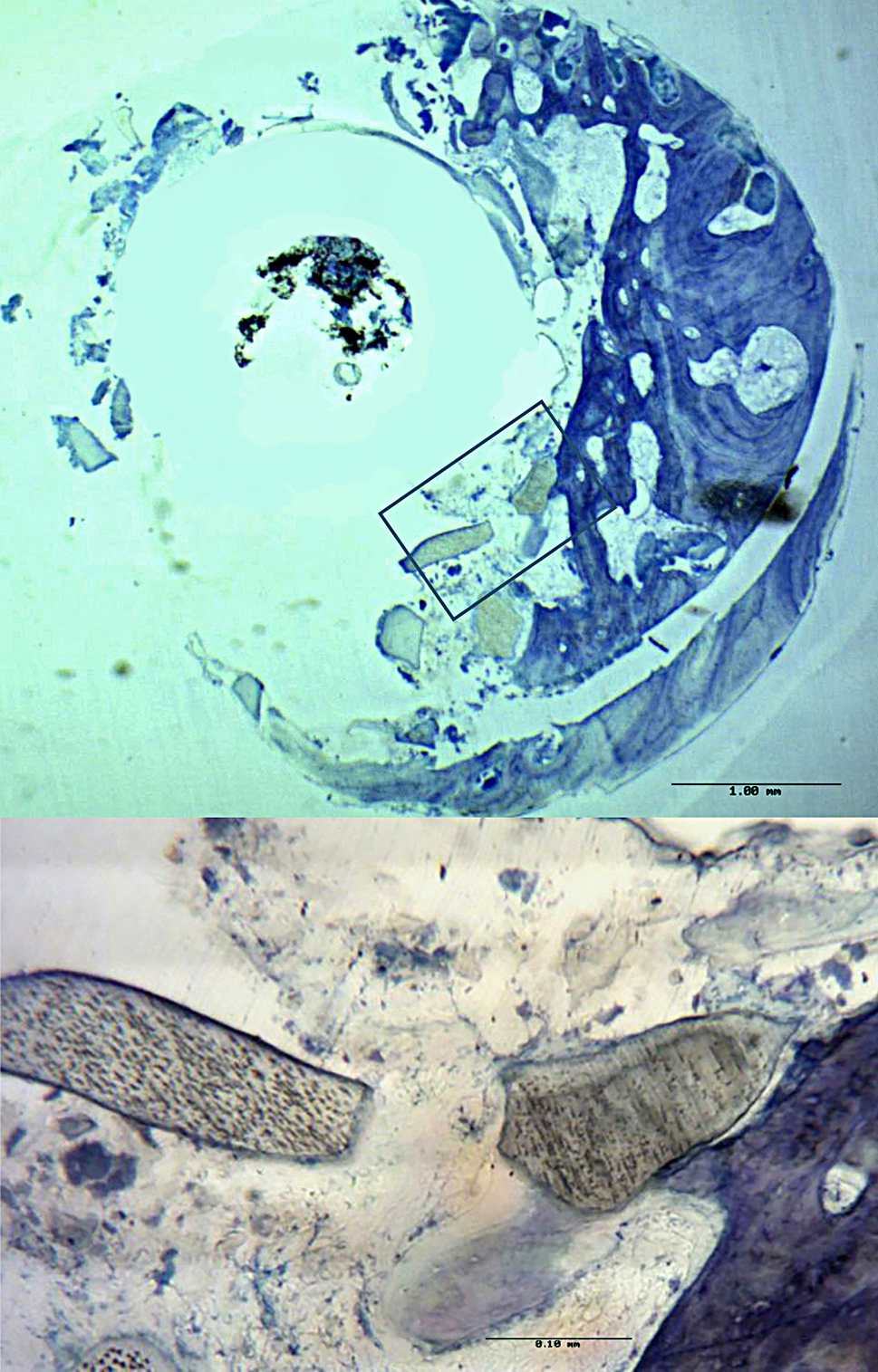
Histological image showing a dentin particle close to osteoblasts. Legend: Cross-section. ADM side. Toluidine blue staining. 3x magnification In the magnified images (x6) showing a dentin particle, with newly formed bone on the right and bone forming below, and the presence of a front of osteoblasts

Representative images are shown in Figs. 4 and 5. In many cases, dentin particles were in close contact with new bone tissue, and even completely embedded in it and in direct contact with the bone (Fig. 4). Further, these dentin particles were associated with fronts of osteoblasts (Fig. 5). Taken together, these findings can be considered indicative of the potential of the dentin-based material used in our patients to stimulate the formation of new bone in alveolar sockets.

## Discussion

The objective of this split-mouth study was to compare the use of fresh ground dentin (ADM) as a filler with that of blood clot stabilisation alone in terms of alveolar ridge preservation. To this end, we radiologically assessed the size of alveolar sockets and bone density around the sockets (in HUs) up to 16 weeks and compared changes in these variables between the groups. Overall, the results support our hypothesis in that they suggest less alveolar ridge remodelling when dentin material was used, in vertical and horizontal radiographic measurements. Further, histological findings suggest very good adaptation with surrounding bone tissue.

Clinical, radiological, and histological studies have shown that, after tooth removal, there is resorption of the external or buccal bone wall (“bundle bone”), it being a periodontal structure. Further, remodelling of the alveolar wall results in some extent of change in the size (both height and width) of the crest [1–3]. Often, effective minimisation of this remodelling becomes the determinant of treatment success in terms of aesthetics and function, or at least, a key factor that influences how much treatment is needed to achieve our goals. As described in the literature [24], the inorganic composition of dentin is very similar to that of bone. For this reason, several authors have focused on dentin as a potential bone substitute. The idea of being able to use fresh ground autologous dentin as a filler material opens the door to an affordable way to preserve the alveolar ridge after tooth extraction surgery.

A process of alveolar socket remodelling occurs after tooth removal and has been reported to be most marked within the first 2 months. A meta-analysis including 20 studies found, based on radiographic measurements, different amounts of shrinkage in different directions, with sockets shrinking in horizontal, vertical, and mid-lingual directions respectively by an estimated 2.54, 1.65, and 0.87 mm at non-molar sites and 3.61, 1.46, and 1.20 mm at molar sites in the first 2 to 9 months after tooth extraction [38]. In relation to this, we found considerable decreases over the study period in most of the dimensions considered (HL, VL, VL-BBC1, and VL-BBC3), in both the ADM group and controls, indicating that it is not possible to maintain the entire pre-extraction alveolar socket volume. Nonetheless, this does not mean that the filler material used does not help achieve the goal of socket preservation. The distance HL, reflecting the total width of the alveolar socket, had decreased less at both follow-ups when dentin particles had been used, with mean reductions of just 0.71 mm at W8 and 1.07 mm at W16 in the ADM group, compared to 1.56 and 2.14 mm respectively in controls. Although differences did not reach significance, the effect size was moderate and the interaction between groups was highly significant. A similar tendency was found for other horizontal dimensions measured. Specifically, we observed mean decreases in VL-BBC1 of 0.51 mm in the ADM group and 0.92 mm in controls at W8, and 0.6 and 1.32 mm, respectively, at W16, and in VL-BBC3 of 0.28 mm in the ADM group and 0.75 mm in controls at W8, and 0.43 and 1.02 mm, respectively, at W16. These measurements correspond to mean decreases in alveolar socket width of approximately 9.1% in the experimental group compared to 19.7% in controls at W8, and 12.8% and 26%, respectively, at W16. Based on this analysis, there seemed to be less shrinkage in alveolar socket width when we used recently ground autologous dentin, though the differences did not reach significance, likely due to the sample size. Further, the decreases in width observed in the experimental group compare very favourably with the pooled data from the aforementioned meta-analysis [38], which found mean alveolar bone shrinkage of 2.54 mm horizontally after removal of non-molar teeth.

Our study builds on the work started by the research group of the Master’s in Oral Surgery, Implant Dentistry, and Periodontics research group at the University of León, in particular, a pilot study on the use of ADM for alveolar grafting after tooth extraction [33]. In line with this pilot study, we also observed decreases in the height of the alveolar socket in the experimental group (at W8, 0.9 vs. 1.12 mm in controls, and at W16, 1.21 vs. 1.3 mm in controls), though somewhat smaller and again the differences and interactions did not reach significance. At the last follow-up (W16), the effect size was moderate, however, which could be considered suggestive of less shrinkage in the experimental group at this time point. It should be noted that the extent of bone volumetric changes after tooth extraction varies depending on patient-related characteristics including gingival biotype, buccal bone plate thickness and any pre-existing buccal bone plate defects. Specifically, in a review, Chappuis et al. found that the impact of bone resorption was much more marked in the case of thin (< 1 mm) than thick (> 1 mm) bone walls (7.5 vs. 1.1 mm) [39]. In future research, it would be interesting to gather more data on these potential explanatory factors.

Regarding our analysis of bone density around the alveolar socket, the high statistical significance (of both differences and interactions) indicates that the sockets treated with ADM had greater bone density in HUs than those that received the control treatment, regardless of the time point and third of the socket analysed. This is likely attributable to the presence of dentin particles themselves, as they tend not to be reabsorbed and have a greater density than bone [40]. Nonetheless, the small amount of shrinkage observed in the ADM group at W8 suggests that, if there is some bone remodelling, there is bone growth after the dentin graft, the bone density increasing again by W16. This observation is consistent with the histological analysis in which we have been able to observe particles of dentin in close contact with bone tissue, and even cell proliferation around dentin particles (Fig. 4). These histological findings are in line with previous research [27, 28] that has attributed demineralised dentin with osteoconductive properties due to the presence of BMPs which can induce new bone growth. Based on our histological analysis, we can state that there is not only no evidence of foreign body reaction or signs of inflammation due to the presence of fresh ground autologous dentin particles (ADM), in agreement with previous studies [32, 41, 42] but also an affinity between these particles and the remaining bone tissue, as observed by Tanoue et al. [43].

We recognise that this study has several limitations. In particular, the researchers were aware of the need to ensure that patients were in the same position for each imaging scan used to obtain comparable socket measurements. To this end, we standardised the procedure, always using the same positioning guides and anatomic references to position patients and using the same reference points on the images for measuring dimensions. Nonetheless, it is not possible to guarantee exactly the same alignment for each scan, and hence, potential differences in position, orientation and/or inclination between imaging sessions remain a limitation of the study. A more general limitation is that the sample studied was relatively small, attributable to the strict selection criteria applied and the requirement for follow-up. In the future, efforts should be made to obtain a larger sample, to confirm the results, especially in the case of variables found to be close to significance and thereby provide a sound basis for our ongoing research concerning implant behaviour in this type of bone socket. Thirdly, the sampling was carried out at a single centre and only included patients between 21 and 62 years of age, and the results cannot be generalised to other populations. Further, the selection criteria used implied the selection of alveolar extraction sockets of single-rooted teeth with intact bone walls, and hence, our results only apply to this type of case, and in particular, cannot be extrapolated to teeth with multiple roots with dehiscence or holes in the alveolar cortical bone.

Recognising the limitations of this study, and considering the good radiological findings together with the promising histological observations, we decided that further research was warranted and have launched a clinical trial (clinicaltrials.gov identifier: NCT06226116 [01/17/2024]). Specifically, in this ongoing trial with a larger sample, we are seeking to explore the behaviour of experimental alveolar implants in contact with ADM in detail, with both radiological analysis of the bone density in a radius of 0.25 mm around the implant site and histological analysis including assessment of bone-implant contact.

## Conclusion

Taken together, the reduction in both horizontal and vertical bone remodelling in the study group at the radiographic level and the high bone density (in HU) indicate that dentin behaves as an ideal slow resorption material for alveolar ridge preservation. Moreover, histological observations suggest that it is fully biocompatible. More research is required to help determine whether this material can be considered the gold standard in alveolar bone regeneration due to its apparent inductive properties.

## Data Availability

The data that support the findings of this study are stored on a password protected database at the University of Léon and are available from the corresponding author upon reasonable request.

## Abbreviations

ADM: Autologous dental material
BMP: Bone morphogenetic protein
CBCT: Cone beam computed tomography
HL: Socket width, measured horizontally from the lingual alveolar crest to a perpendicular line from the outer part of the vestibular cortex
HL-BBC: Difference in height between the lingual and buccal alveolar crests, measured as the minimum distance from a line drawn horizontally at the lingual alveolar crest to a perpendicular line from the outer part of the vestibular cortex
HUs: Hounsfield units
VL: Socket height, measured vertically along the long axis of the missing tooth from the fundus of the socket to the lingual alveolar crest
VL-BBC1, VL-BBC3, and VL-BBC5: half-widths from a vertical line along the long axis of the missing tooth to the buccal alveolar crest at 1, 3, and 5 mm below the crest
W: Week
